# Advanced Comparison of Phased Array and X-rays in the Inspection of Metallic Welding

**DOI:** 10.3390/ma15207108

**Published:** 2022-10-13

**Authors:** José Alonso, Santiago Pavón, Juan Vidal, Manuel Delgado

**Affiliations:** 1 Department of Applied Physics, Centro Andaluz Superior de Estudios Marinos, University of Cádiz, 11510 Puerto Real, Cádiz, Spain; 2 Department of Ship Building, Centro Andaluz Superior de Estudios Marinos, University of Cádiz, 11510 Puerto Real, Cádiz, Spain; 3 Department of Ship Building, Quality Inspection, Navantia San Fernando, Carretera la Carraca s/n, 11100 San Fernando, Cádiz, Spain

**Keywords:** phased array, X-rays, metallic welding, nondestructive inspection

## Abstract

The most common nondestructive weld inspection technique is X-rays and, since a few years ago, the ultrasound-based phased array. Their comparison has been done from the top view of both, with the result that the phased array is much more efficient in discovering flaws. From the last studies of the authors, a welding flaw can be three-dimensionally reconstructed from the sectorial phased array information. The same methodology is applied to compare quantitatively X-rays and phased array on 15 metal inert/active (MIG/MAG) welding specimens covering pores, slag intrusion and cracks. The results can be summarized in the correlation of the top views and in the correlation profiles between the X-ray top-view and the reconstructed top-view at the depths from phased array in the weld. The maximum correlation is the depth when the flaw in the X-ray looks like that in the phased array records at some depth, leading to an effective quantitative comparison of X-rays and phased array.

## 1. Introduction

The techniques for welding inspection can be classified as destructive and nondestructive. The first ones imply the mechanic breaks the weldment and the working piece to inspect. The second ones keep the piece and make use of nondestructive techniques (NDT) such as visual inspection, magnetic particles, dye penetrants and advanced instrumental techniques based on ionizing radiation, ultrasounds or electromagnetic fields [1,2,3,4,5].

The NDT based on ionizing radiation is X-rays. This is the first and most used technique for welding inspection as its results allow a very easy documentation in quality reports [6]. The use of X-rays has many disadvantages, such as the isolation of the working piece in a shielded bunker, when possible, the stopping of the work in the area of the inspection because of the reflected ionizing radiation rays, a long exposition time up to the thickness of the material and a poor location of the flaws in a weldment. In addition, X-rays cannot detect many defects when compared to other NDTs. It gives only top-view information.

The NDTs based on ultrasounds are the TOFD (time-of-flight diffraction), PA (phased array) and EMAT (electromagnetic acoustic transducer). Although EMAT is based on electromagnetic induction, this is the physical mechanism to generate the ultrasounds for inspection in the metallic piece; hence, it can be considered in the group of ultrasound-based NDTs [1,2,3,4,5]. The classification societies accepted PA as an NDT a few years ago. Ultrasound-based techniques have many advantages. The ultrasounds are innocuous for health and the work can continue in the place of inspection, taking a short time for inspection. The disadvantages are that the instruments are more expensive than those for X-rays and training is needed.

The NDTs have been combined with mathematical morphology, neural networks, fuzzy logic, wavelets and stereo vision for automatic flaw identification but using the top-view data [7,8,9,10,11,12,13,14,15,16,17,18,19] with very advanced tools for computation. The comparisons between NDT uses are the determination of alternative configuration for the equipment [20], the comparison of the acoustical echoes [21] or testing the capability of the different NDTs when working on simple or complex geometries [22]. In addition, an NDT can be combined with a destructive technique to study a flaw completely [23]. A mode detailed and a qualitative comparison based on experiments on different working pieces among X-rays, PA and time-of-flight diffraction can be found in [24].

It is well-known that PA is more efficient than X-rays, detecting more flaws in the same weldment, needing less time to inspect without the risk of ionizing radiation. When an X-ray and a PA inspection of the same weldment are available, the only way for comparing them is through their top views. A natural question is how much the information of the X-rays match with the sectorial views from the PA. In other words, at which depth of the weldment is the top view of the X-ray equivalent to the information from PA?

The authors compare the results of X-rays and PA inspections in 15 weldment specimens, 5 with pores, 5 with slag intrusions and 5 with cracks. All of them were made by metal inert/active gas (MIG/MAG) welding. The comparison is first carried out in a qualitative way to identify flaws. Then, a quantitative comparison to compute how similar the top views from X-rays and PA are and to estimate the depth in the weldment where the PA inspection corresponds to the X-ray results. This is the main contribution of this study and most of the methodology follows [25].

Results are summarized as: (i) a methodology for the quantitative intercomparison of NDTs; (ii) the computation of the correlation between the flaw signatures from X-rays and PA; and (iii) the computation of the depth in the weldment closest to the results of the X-ray top view.

## 2. Materials and Methods

### 2.1. Weldment Specimens

One hundred probes of steel-grade S275JR+N DIN EN 10025, common in shipbuilding, were made under controlled testing weldments at the welding facilities of the School of Naval and Ocean Engineering at the University of Cádiz. The dimensions of the specimen were 250 mm long, 300 mm wide and 12 mm thick. The mechanical properties and chemical composition of the material are presented in Table 1.

**Figure 1:**
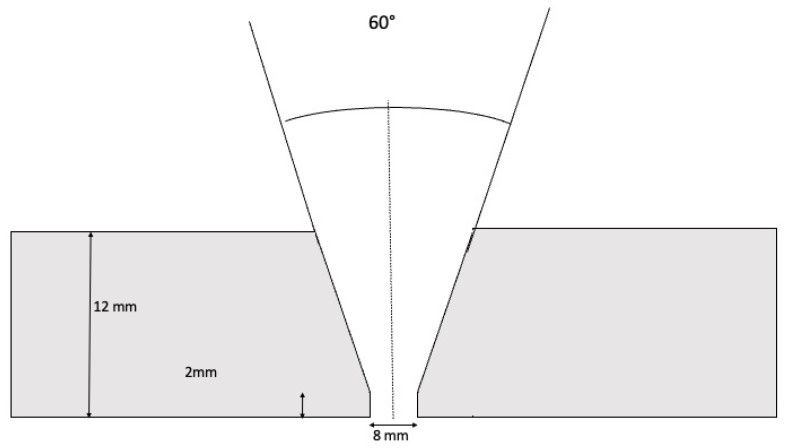
Scheme of a weld specimen.

The plates of the specimen had a 30° chamfer in one of the sides of 250 mm long and a MIG/MAG welding with a ceramic backing and a final cleaning was carried out. All weldments were visually inspected. The schematic cross-section is shown in Figure 1, and it is basically the same as that used in [25].

The flux core arc welding (FCAW) used FLUXOFIL 14HD of 1.2 mm in diameter. Its mechanical properties and chemical composition are presented in Table 2.

Four groups with the same numbers of probes were considered: 25 with no error (G-probes), 25 with pores (F1-probes), 25 with slag intrusion (F2-probes) and 25 with cracks or fissures (F3-probes). The error-free specimens allow the determination of the noise level of the NDT in the material. Among them, five specimens with flaws where selected for the purposes of this study.

The different defects were induced by changing the welding conditions. The improper gas shielding induces pores. If some slag from previous welds is added and the weld carries on, the new material has the same properties as the added slag and it produces slag intrusion. When some very short copper or steel wires, with a fusion point much lower than the temperature of the torch, are added, a crack is induced. The qualitative analysis of the specimens can be found in [25].

The welding process had seven runs. The first one is a weld root cord made with 200 A current, 24 V voltage and 18 l/min of gas flow. Now a wide weld filler bead was applied with 260 A, 27 V and the same gas shield. This is the point to act if a defect must be induced. The next two runs are for a thin weld filler bead with the same conditions of the wide weld filler bead. Finally, three more runs for welding combing cord with 210 A, 26 V and the same gas shielding. All specimens were performed by a professional welder technician at the School of Naval and Ocean Engineering of the University of Cádiz [25]. All the specimens were inspected using PA and two specialists and a level III inspector in weld quality classified the different defects.

### 2.2. Phased Array Ultrasound Inspection and Data Preprocessing

Among the nondestructive ultrasound-based techniques for weld inspection, the phased array (PA) is the most advanced. The beams with multiple angles and different focal depths, or focal laws, enables one to carry out a full inspection consisting of a top view and a set of sectorial views in just one run [19]. The array of single housed transducer and sampling windows are sequentially activated or phased. The transducer can have from 16 to 256 individual micro-transducers. The geometry may be square, rectangular or rounded, and the working frequencies range from 1 to 10 MHz.

The PA technology presents many advantages and some disadvantages [19]. The advantages include: (i) it is possible to inspect complex geometries [23]; (ii) it can be used on metallic [21] and composite materials [19]; (iii) it is possible to inspect high-temperature specimen; (iv) it is faster for inspection than X-rays and other nondestructive techniques; and (v) it is capable of detecting more flaws compared to other NDTs. The technique can also be combined with destructive assays [23]. However, the PA requires permanent physical contact between the transducer and the weldment by means of a coupling gel. This means that the inspection of a long weldment can be quite uncomfortable and cumbersome because of the amount of coupling gel to use.

The ultrasound inspections were carried out by means of a phased array Sonatest Veo+ system [26] with a 64 pulser/receiver transducer X3A-5M64E-0.6X10 (X3AW-N55S) at 125 MHz with high resolution and performance. The equipment provides 55 different focal laws (beams) of 32 elements, covering a range of 55 angles from 45° to 72° every 0.5°, using a 5 MHz excitation frequency with a 64 elements transducer. The configuration was set to ensure the maximum weldment coverage with two runs, to the right and to the left. The transducer was equipped with a wheel and the C-scans were recorded every millimeter. The option of TCG (time-corrected gain) was also selected to ensure a homogeneous response across the entire area of the sectional view, as well the usual calibration. This means that the instrumental response was the same in any stage of the inspection. The setup of the equipment was carried out by InnerSpec [25].

**Figure 2:**
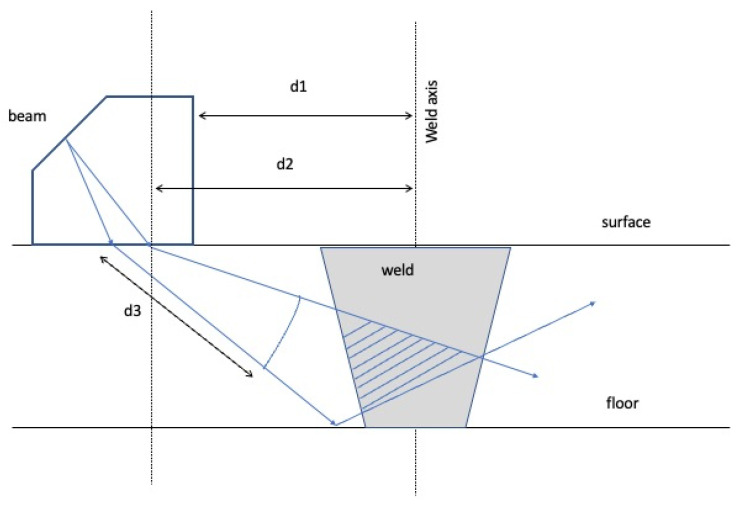
Geometry of the PA inspection.

**Figure 3:**
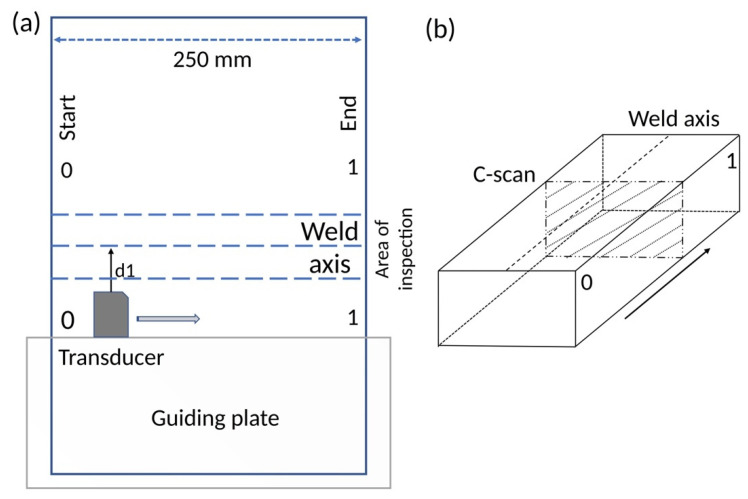
(**a**) Scheme of the welding inspection detailing the weld axis and the position of the transducer on the guiding plate; (**b**) Position of the C-scans and the weld axis.

The geometry of the inspection is presented in Figure 2. Fixing the reference frame in the axis of the joint, the distance to the transducer was constant (d1 = 20 mm). Each one of the 55 beams has its own distance between the incidence point and the axis of the joint (d2). The measurements start at a distance d3 from the incidence point with an increment of 0.052 mm, being 576 [25]. A metallic guide was made and fixed to each specimen being inspected (Figure 3a) to keep the distance of the transductor–weld axis constant.

An inspection is composed of two runs, at the left and the right of the weldment, starting at 0 mark (Figure 3a). Both were combined to obtain a view of the whole joint. A wheel to code the distance was attached to the transducer and programmed to read every millimeter along the 250 mm of the weld axis (Figure 3a). Every millimeter a C-scan, a sectorial view (Figure 3b), was taken. So, the minimum size flaw that can be detected is 1 mm in the *x*-axis and about 0.1 mm (the double of 0.052 mm) in the other two directions. The datasets, the top and sectorial views, were exported to comma-separated value files and the final dataset for each probe consisted of a top view and 250 sectorial sections [25].

The raw data were processed with several FORTRAN codes developed by the authors, as in [25]. The code processes the top views by eliminating the first and the last sections to avoid border effects. The sectorial view datasets were interpolated using the kriging method [27] with a previous estimation of the variograms, spherical for all cases, being a quite complex and delicate step. The final dataset consisted of 250 C-scans with 480 depth levels in the 12 mm thickness of the plates. The information from the interpolated C-scans can be used by slicing; this is getting the data at a fixed distance from the surface. From here and after, the depth in a weldment will be expressed as the number of slices from its surface. The thickness of each one is 12/480 mm to keep the above sampling distance.

### 2.3. X-ray and Image Processing

The X-ray inspections were carried out using a D5806 model with a radioactive source of iridium with an activity of 45.2 Ci. The source size is 2.7 × 2.55 mm. The radiographic plates were a D3 AGFA class with dimensions of 10 × 40 cm. The inspection follows the UNE-EN ISO 17636-1:2013 and the evaluation standard was UNE-EN ISO 10675-1:2017. The radiographic technical procedure was OCA Global “IT-CI-PX-17 Rev03 01/06/2019”. The distance focus-object was 388 mm and focus-film 400 mm. The exposure time was 4 min and 55 s. X-ray inspections were carried out in the facilities of OCA Global at Cádiz, Spain.

The X-ray imagery was digitalized and the 8-bit panchromatic gray scale digital images were submitted to an expansion of contrast and a high pass filter to highlight the flaws. This is enough to improve the quality of the X-ray images.

### 2.4. Data Processing

Once the preprocessing is achieved, the digital images, the numerical matrices, of the X-ray and phase array top views and the interpolated phased array sectorial views are ready to be processed. The first step is the identification and isolation of the flaws from the top-view imagery. This is carried out by identifying the flaw in both top views, checking for the coordinates in the numerical matrices and isolating the corresponding interval. When needed, the dimensions of the matrices were made the same by subsampling. The correlation was computed by means of the Pearson coefficient for linear correlation:

(1) $$r=\frac{Cov\left(x,y\right)}{\sigma_{x}\sigma_{y}}$$

where *x* is the set of brightness digital levels from the PA image, and *y* is the corresponding to the X-ray image. This quantifies the relationship between the X-rays and PA top-view signatures.

It is well-known that X-rays are not so efficient as phased array for the detection of welding flaws. To compute the relationship between the inspections of X-rays and the top view, the slice (see Section 2.2), at any depth from phased array, the same correlation analysis was applied between the X-ray image and the 480 computed slices, one by one. The result is a profile of correlation. Only the first 300 slices were considered because below that level is the root cord. The highest absolute value gives the depth where the signature of the flaw in the phased array seems close to the X-ray top view.

The process is quite difficult and time consuming for the researcher because the different data came with different spatial resolution and the inspections cover different areas. Fortunately, the spectral resolution is the same, 8 bits, in the panchromatic images. The data processing was carried out using MatLab.

## 3. Results and Discussion

### 3.1. Flaws Identification from B-Scans

**Figure 4:**
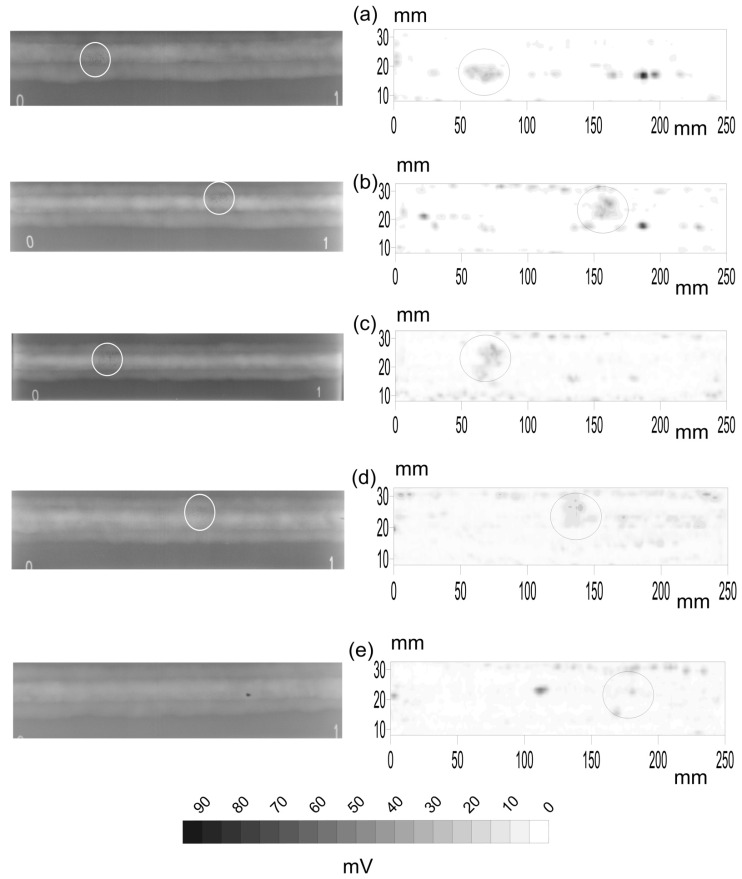
X-ray (**left**) and phased array inspection (**right**) of five specimens, (**a**–**e**), with pores. Circles point out the coincident pores. The length of the specimen is 250 mm and their width is 30 mm with the axis of the weldment in the middle. The small numbers 0 and 1 in the X-rays images were added when inspected.

**Figure 5:**
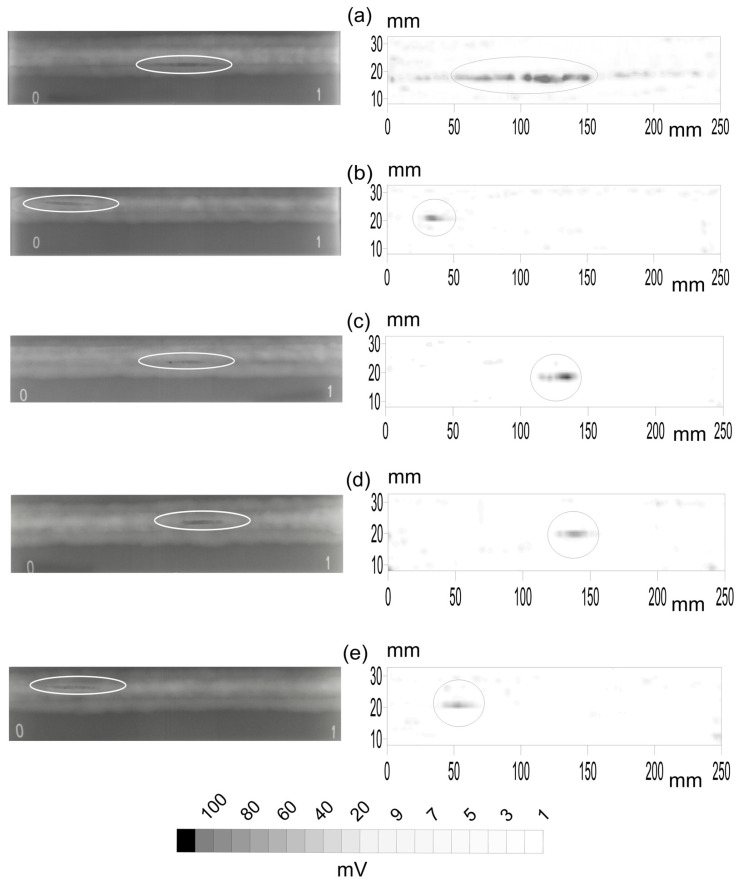
X-ray (**left**) and phased array inspection (**right**) of five specimens, (**a**–**e**), with slag intrusion. The length of the specimen is 250 mm and their width is 30 mm with the axis of the weldment in the middle. The small numbers 0 and 1 in the X-rays images were added when inspected.

**Figure 6:**
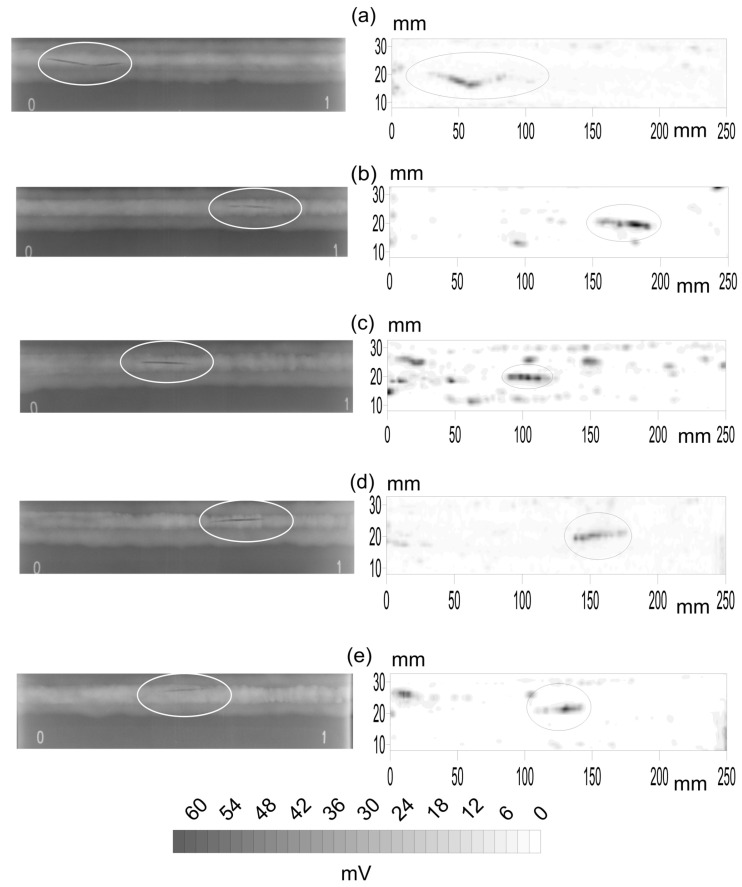
X-ray (**left**) and phased array inspection (**right**) of five specimens, (**a**–**e**), with cracks. The length of the specimen is 250 mm and their width is 30 mm with the axis of the weldment in the middle. The small numbers 0 and 1 in the X-rays images were added when inspected.

The first stage is the comparison of the X-ray and PA top views. Among all the available probes, five specimens with pores, five with slag intrusion and five with cracks were selected to be radiated. The X-ray gamma-graphic and the corresponding B-scan of the PA inspections for the different flaws are presented in Figure 4 for pores, Figure 5 for slag intrusion and Figure 6 for cracks.

Concerning the case of specimens with pores (Figure 4), the X-ray detects only one group of pores while PA can detect more flaws. In fact, there are cases in which the PA has plenty of signals while only one is seen in X-rays. The circles detail the group of pores in both images for the five specimens. Now it is possible to see at what it was pointed in Section 2.4 about the difficulty of the data processing at the time of determining the coordinates of the flaw in both images.

**Figure 7:**
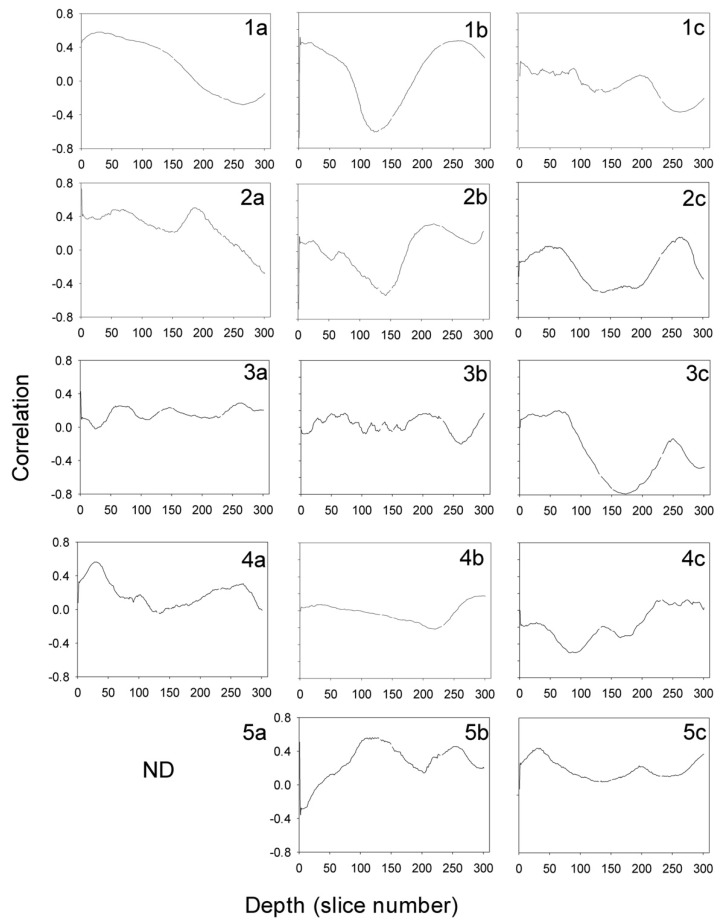
Profiles of the correlation between the X-ray B-scans and the interpolated phased array B−scans from 1 to 300 slices in depth. The first datum is the correlation between the top views of X−ray and PA inspections. Column (**1**–**5a**) is for the specimens with pores, column (**1**–**5b**) for slag intrusion and column (**1**–**5c**) for cracks. ND stands for ‘no detected’.

The linear relationship between the signatures of the flaw in both images is the first correlation coefficient in the plots of Figure 7. The correlations for pores, Figure 7 column a, are 0.455 (1a), 0.730 (2a), 0.426 (3a), 0.078 (4a) and 0.0 (5a). The results for the last specimen are not presented in X-rays. This will be fully explained in the next subsections. The high value of the correlations means that pores have a clear signature in the depth-integrated view, the top view, of the X-rays and PA.

The same happens with the slag intrusions (Figure 5). The ellipses point to the analyzed flaws. Now, an artifact can be observed in the X-ray B-scans as a continuous line in the welding axis. This can be easily guessed by the experiment eye of the welding quality inspectors. In general, the correlation in Figure 7 (column b) is quite high: −0.681 (1b), −0.683 (2b), −0.006 (3b), −0.032 (4b) and 0.508 (5b), except in the specimens c and d.

Finally, the X-ray and PA inspections of the specimens with cracks are presented in Figure 6. The cracks are pointed out as before and easily guessed. The PA inspections show plenty of other flaws that will not be studied here. Nevertheless, most of them are not present in the X-rays images but in the PAs. Four of the five specimens, Figure 7 (column c), present correlations close to zero (0.049 (1c), −0.318 (2c), 0.0 (3c), 0.0 (4c) and −0.677 (5c)), except the last specimen.

### 3.2. X-ray and PA B-Scan in Depth

The second step in the comparison is the computation of the depth in the weldment where the X-ray is really representative or seems like a top view in the depth of the PA. Following the computational processes of Section 2.4, the coordinates of the flaw in each inspection were determined. This was quite difficult because in many cases the flaw is detected in the X-ray but not in the first slice of the phased array. In those cases, a search in depth was carried out to find the flaw. Then, the coordinates are determined, the corresponding region of the matrices isolated and the data subsampled to make then have the same dimensions. The Pearson linear regression coefficient is then computed. This algorithm was made in MatLab.

The profiles in the weldment of the Pearson coefficient for linear relationship are presented in Figure 7. In the case of pores, Figure 7(1a) presents a plate until the depth 100 approximately. This means that pores follow the spatial structure of the plain signature in X-rays. After that, the correlation falls. Figure 7(2a) shows two maxima, where the pores are grouped, falling after depth 200. On the other hand, Figure 7(3a) is plain with a correlation close to zero. This means that pores and the unavoidable noise are mixed. This flaw is difficult to see in the X-ray B-scan in Figure 4. However, a clear correlation is seen in Figure 7(4a) in a very shallow depth in the weldment. This is also very clear in Figure 4. Finally, the profile in depth of the correlation in Figure 7(5a) is not presented because the flaw is not seen in X-rays but in PA (Figure 4).

The correlation profiles in the slag intrusion are presented in column b. Now the behavior is very different. Figure 7(1b) shows a very defined peak at a depth of 110, where the slag was added, and it clearly appears in Figure 5. Figure 7(2b) shows some noise, small amplitude oscillations, and a peak at 150. However, Figure 7(3b) shows a very noisy profile and is close to zero. These are in agreement with the tiny signal in the X-ray B-scan of Figure 5. More or less the same happens with Figure 7(4b). Two peaks are observed in Figure 7(5b).

Concerning the specimens with cracks, Figure 7(1c) shows very low correlations, but the signal is extremely clear in Figure 6. This is because the crack gets into the weldment and its shape is distributed in depth non-uniformly. Figure 7(2c,2d) show a zero correlation plate at the beginning and a peak at about depth 170. These signals are very clear in Figure 6. Figure 7(4c) shows the maximum depth of about 100 and the rest of the profile is zero, locating the flaw very well. Finally, Figure 7(5c) points out the crack at a depth of about 140, but because the peak is wide, the crack is deep in the weldment.

### 3.3. Three-Dimensional Reconstruction and Correlation

**Figure 8:**
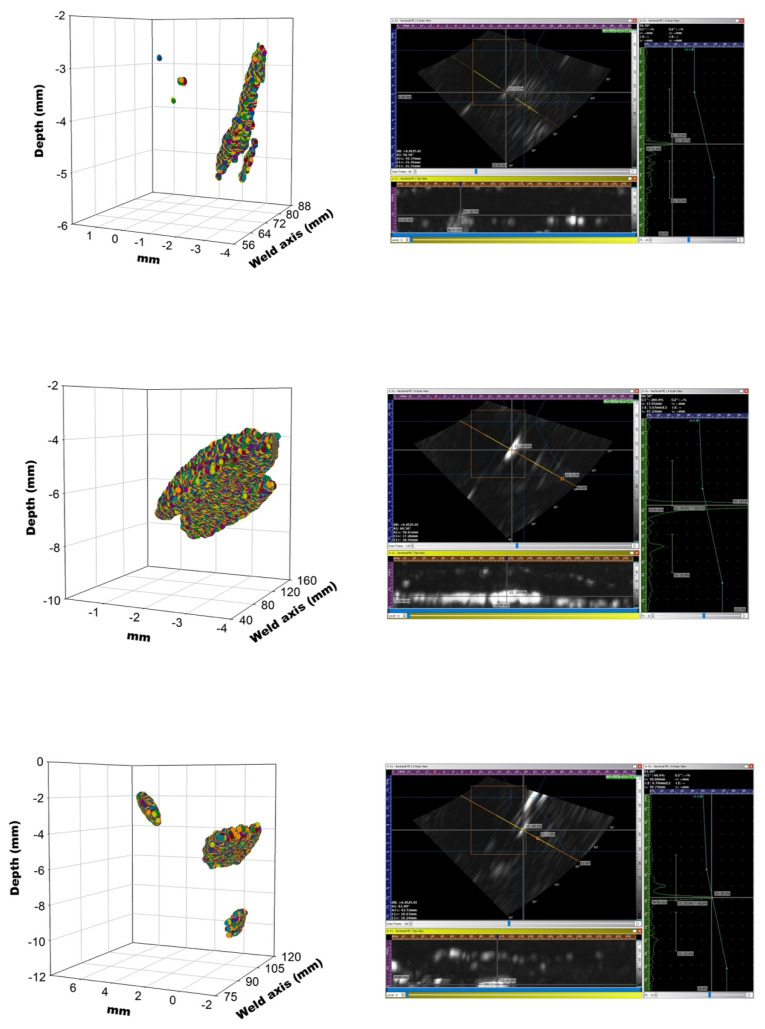
Three-dimensional reconstruction (**left**) and PA inspection (**right**) of case 1a (**top**), 1b (**middle**) and 3c (**bottom**) with reference to Figure 7.

The three-dimensional reconstruction of the flaws for the considered specimens of Figure 4, Figure 5 and Figure 6 and the correlations shown in Figure 7 were carried out. Three of them were selected and they are presented in Figure 8 together with the screen Sonatest Veo+ phased array.

The vertical structure of the correlation in Figure 7(1a) shows a plateau with a correlation value of about 0.5. The correlation falls at slice 150 approximately, corresponding to a depth of 3.75 mm from the surface. This is in agreement with the three-dimensional reconstruction of Figure 8 (top left) and with the location of the pike of maximum echo intensity in the PA inspection (Figure 8 (top right)). It is quite remarkable that the pores have effect to several depths (Figure 8 (top left)) in the weld, and that is read in the vertical profile of correlation (Figure 7(1a)) and in the PA inspection (Figure 8 (top right)).

The slag inclusion affects between the slices 60 and 150 in the case of the three-dimensional reconstruction of Figure 8 (middle left) that corresponds to Figure 7(1b). The PA inspection shows the peak in Figure 8 (middle right).

Finally, the crack of Figure 7(3c) is reconstructed in Figure 8 (bottom left) and the PA inspection is in Figure 8 (bottom right). Again, a good agreement among all results happens.

There are some cases in Figure 7 in which the correlation is close to zero at all depths. They are easily explained from Figure 8. The X-rays give an integrated signal in the volume of the working piece with a certain flaw signature, but if the defect is not horizontal the top view in depth will show just only part of the defect. Hence, the correlation must fall.

## 4. Conclusions

The above experiments comparing the top views from X-rays and PA, and the top view at any depth from PA with X-rays, lead to the following conclusions:

- The PA is more sensitive to flaws than X-rays.
- Different spatial resolution makes this kind of comparison time consuming.
- The top view from X-rays and PA can give similar information in the case of pores, sometimes in the case of slag intrusion and with some difficulty with cracks. This is shown in the correlation coefficient between the corresponding areas where the surface signature of the defect is seen in both kinds of techniques. From the knowledge of the authors, this is the first time that this kind of quantification was carried out.
- The PA allows the three-dimensional reconstruction of the flaw and, if needed, the computation of its size and volume from the previous methodology of [25].
- The PA allows us to see if a flaw affects only to a depth or to some depths in the weld. In other words, it allows the study of the vertical structures of the defect. This can be especially important with cracks.
- This methodology allows the determination of the depth at which the X-ray inspection is more representative from the computation of the linear coefficient regression, always in a quantitative way, being the first time that this kind of study was carried out.

## Figures and Tables

**Table 1:** Mechanical and chemical properties of the S275JR + N.

Mechanical Properties											
Yield point (ReH MPa)				Tensile strength (ReH MPa)				Elongation (%)			
309				447				31			
Chemical Properties (%)											
C	Mn	Si	S	P	Cr	Ni	Cu	Al	V	N	CE
0.15	0.84	0.18	0.007	0.016	0.03	0.03	0.06	0.026	<0.005	0.005	0.30

**Table 2:** Mechanical properties of FLUXOFIL 14HD.

Mechanical Properties											
Yield point (ReH MPa)				Tensile strength (ReH MPa)				A5 (%)			
530				600				25			
Chemical properties (%)											
C	Mn	Si	S	P	Cr	Nb	Cu	Al	V	Ti	B
0.049	0.07	0.48	0.008	0.007	0.04	0.01	0.05	0.019	0.03	0.093	0.0035

## Data Availability

The raw/processed data required to reproduce these findings cannot be shared at this time as the data also form part of an ongoing study.
